# Splicing of platelet resident pre-mRNAs upon activation by physiological stimuli results in functionally relevant proteome modifications

**DOI:** 10.1038/s41598-017-18985-5

**Published:** 2018-01-11

**Authors:** Giovanni Nassa, Giorgio Giurato, Giovanni Cimmino, Francesca Rizzo, Maria Ravo, Annamaria Salvati, Tuula A. Nyman, Yafeng Zhu, Mattias Vesterlund, Janne Lehtiö, Paolo Golino, Alessandro Weisz, Roberta Tarallo

**Affiliations:** 1 Laboratory of Molecular Medicine and Genomics, Department of Medicine, Surgery and Dentistry “Scuola Medica Salernitana”, University of Salerno, Baronissi, (SA) Italy; 2 Genomix4Life srl, Department of Medicine, Surgery and Dentistry “Scuola Medica Salernitana”, University of Salerno, Baronissi, (SA) Italy; 3 Department of Cardio-Thoracic and Respiratory Sciences, Section of Cardiology, University of Campania “Luigi Vanvitelli”, Naples, Italy; 4 Department of Immunology, Institute of Clinical Medicine, University of Oslo and Rikshospitalet Oslo, Oslo, Norway; 5 Science for Life Laboratory, Department of Oncology-Pathology, Karolinska Institutet, Stockholm, Sweden

## Abstract

Platelet activation triggers thrombus formation in physiological and pathological conditions, such as acute coronary syndromes. Current therapies still fail to prevent thrombotic events in numerous patients, indicating that the mechanisms modulating platelet response during activation need to be clarified. The evidence that platelets are capable of *de novo* protein synthesis in response to stimuli raised the issue of how megakaryocyte-derived mRNAs are regulated in these anucleate cell fragments. Proteogenomics was applied here to investigate this phenomeon in platelets activated *in vitro* with Collagen or Thrombin Receptor Activating Peptide. Combining proteomics and transcriptomics allowed in depth platelet proteome characterization, revealing a significant effect of either stimulus on proteome composition. *In silico* analysis revealed the presence of resident immature RNAs in resting platelets, characterized by retained introns, while unbiased proteogenomics correlated intron removal by RNA splicing with changes on proteome composition upon activation. This allowed identification of a set of transcripts undergoing maturation by intron removal during activation and resulting in accumulation of the corresponding peptides at exon-exon junctions. These results indicate that RNA splicing events occur in platelets during activation and that maturation of specific pre-mRNAs is part of the activation cascade, contributing to a dynamic fine-tuning of the transcriptome.

## Introduction

Platelets are anucleate cytoplasmic fragments, deriving from precursor megakaryocytes, which play key roles in processes such as thrombosis, hemostasis, inflammation, wound healing and angiogenesis. They circulate in the bloodstream in an inactive, resting state and, once activated, are capable of binding to blood vessel walls aggregating and forming thrombi, to prevent excessive bleeding following endothelial damage. Indeed, activation of platelets and the coagulation cascade are major events in intravascular thrombus formation occurring in diseases such as acute coronary syndromes (ACS), peripheral artery diseases (PAD) and stroke^1–3^. Thus, modulation of platelet aggregation and coagulation pathways represents the main therapeutical strategy pursued in the management of thrombotic disorders^4^. In spite of this, current antithrombotic approaches still fail to prevent thrombotic coronary events in a substantial number of patients^5^, indicating that the complex mechanisms modulating platelet response during activation are not completely understood. A critical issue in such cases is the possibility to assess platelet activation extent before tissue damage occurs, i.e. before myocardial necrosis. A better understanding of the processes that characterize platelet activation, and early markers of these processes, are still much sought after as they may be of great clinical relevance^6^. For a long time the proteome endowment of platelets has been considered static. However, it is well known now that these cell-fragments contain both a pool of megakaryocyte-derived mRNAs^7,8^, encoding for different proteins^9–12^, and the complete machinery for *de novo* protein synthesis, making possible dynamic modifications of protein expression in mature platelets^12^. We recently reported that activating stimuli induce proteome reorganization *via* microRNA (miRNA) modulation^13^, suggesting that platelets contain molecular machineries for post-transcriptional regulation of mRNA activity, a result confirmed also by other studies^14–16^.

Evidence that platelets are capable of *de novo* protein synthesis^17^ also raised the issue of whether resident mRNAs are regulated in circulating platelets and, if so, why. Interestingly, it has been shown that platelets contain a broad spectrum of RNA molecules, including, in addition to mRNAs and miRNAs, also pre-mRNAs and a role of mRNA splicing in regulation of platelet protein synthesis has been proposed^18,19^. Indeed, Denis *et al*.^18^ identified pre-mRNA splicing as a misplaced nuclear process that can occur in platelets, where critical splicing factors are present. The presence of partially spliced transcripts, carrying retained introns, is known to be widespread in mammalian cells, where their presence has been suggested to be a biologically relevant phenomenon that contribute to fine-tuning of the transcriptome preventing, for example, accumulation of unwanted gene products during development or differentiation^20^. Recent evidences, indicating the importance of intron retention/removal as a mechanism for post-transcriptional regulation of gene expression that can go awry in cancer cells^21^, suggested that the mechanism(s) controlling intron retention might represent novel therapeutic targets^22^.

The present study aimed at investigating on a global scale whether changes in the maturation of specific RNAs by non-canonical splicing events occur in platelets upon activation by physiological stimuli, and if this may result in modulation of protein expression. To this end, we performed an in-depth proteome analysis of resting platelets using peptide level high-resolution isoelectric focusing and reverse phase fractionation^23^, followed by mass spectrometry (MS) analysis (HiRIEF LC-MS/MS). Proteome quantification was performed by isobaric labelling (TMT), leading to a comprehensive mapping of the platelet proteome, with identification of >5000 proteins. Subsequently, RNA-Seq provided a catalog of partially spliced mRNAs in resting platelets and showed how retained introns are specifically removed upon activation, to generate mature mRNA molecules. This last result was reinforced by comparison of proteomics and transcriptomics data, showing coherence between intron removal events and accumulation of peptides mapping on the corresponding exon/exon junctions. This aproach allowed the identification of seven transcripts, specifically involved in platelet shape changes, showing reduced intron retention and higher peptide representation at exon-exon junctions in activated *vs* resting platelets.

Overall these results indicate that during platelet activation a large pool of resident pre-mRNAs undergo splicing, resulting in several cases in accumulation of the encoded proteins. We propose that maturation of activation-modulated transcripts represent a novel mechanism for control of platelet functions and may be used as early marker of their activation in thrombotic diseases.

## Results

### Platelet proteome profiling

**Figure 1:**
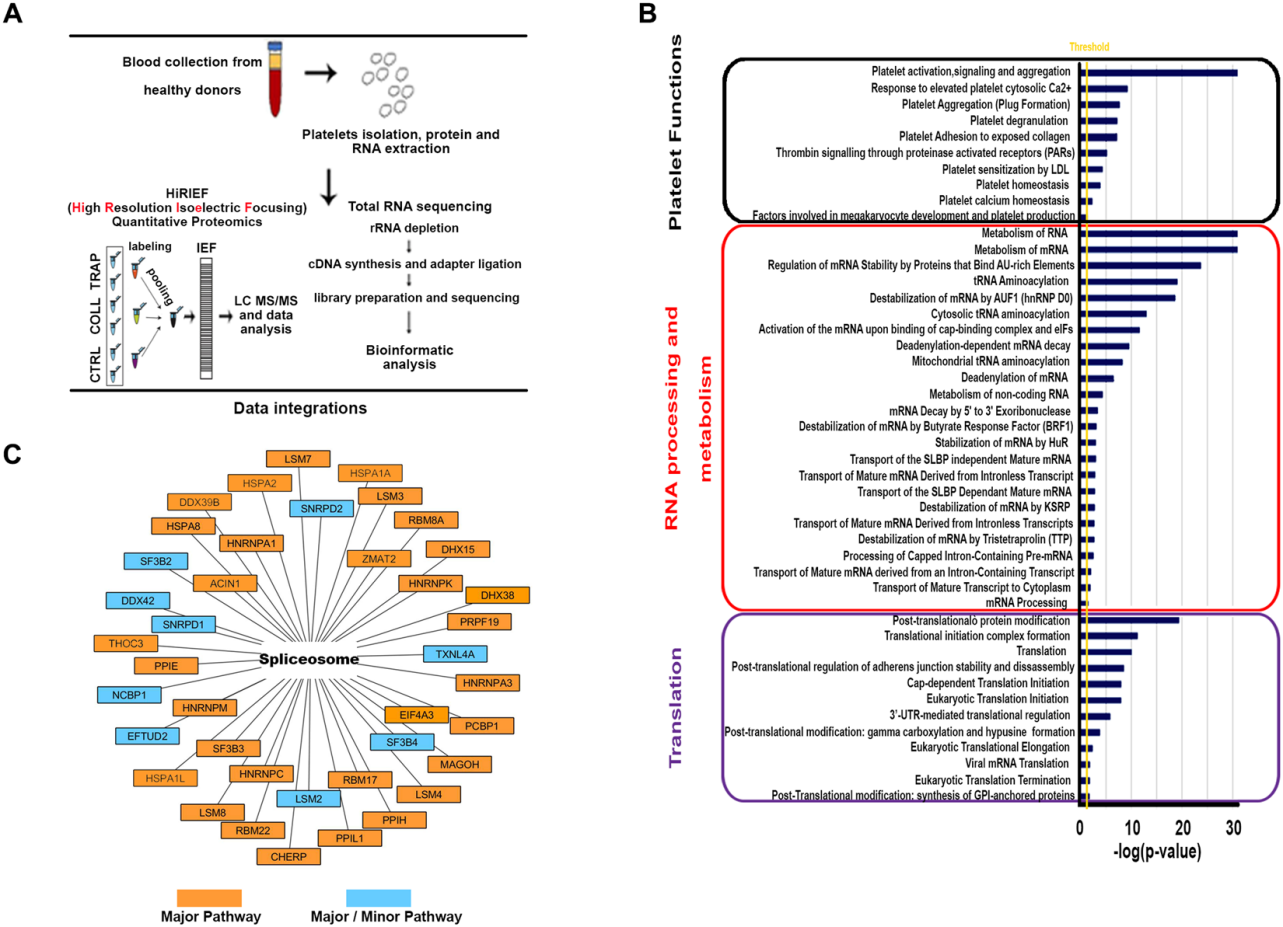
Platelet proteome profiling. (**A**) Schematic representation of the workflow implemented. (**B**) Functional analysis performed with panther tool, showing the most represented biological processes among the identified platelet proteins. (**C**) Proteins of the spliceosome machinery identified in resting platelets by HiRIEF. Major/minor splicing pathway components, acconding to the annotations in the Interactome database, are shown.

**Figure 2:**
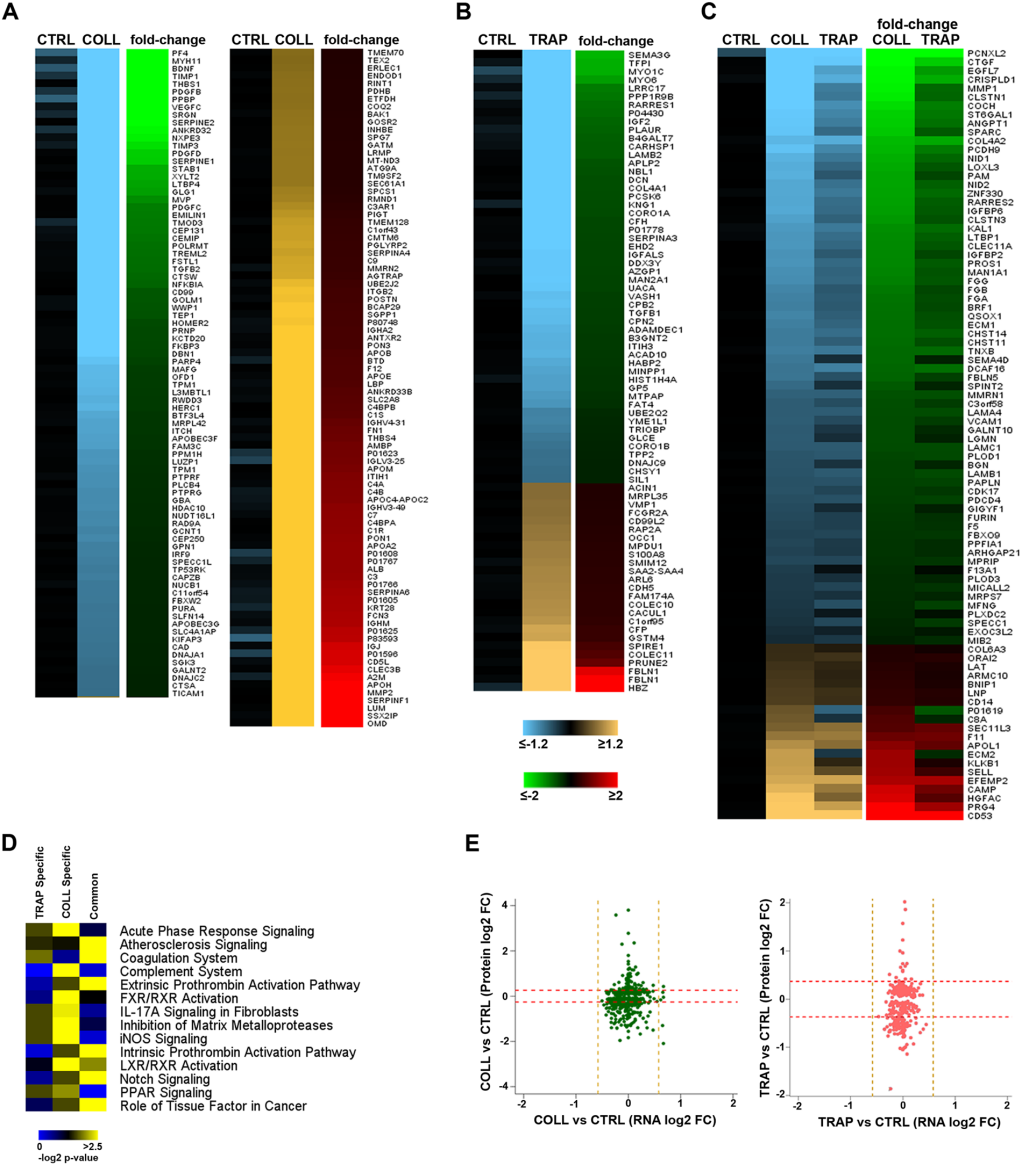
Quantitative profiling of platelet proteome before and after *ex vivo* activation. Heatmaps displaying platelet proteins responsive (FDR < 0.01) to COLL (**A**), TRAP (**B**) or to either agonist (**C**). Average LFQ intensity values obtained from analysis three independent replicate samples are shown in a yellow-blue scale and the corresponding fold-change after activation in red-green scale. Proteins are ranked from the most down-regulated (top) to the most up-regulated ones (bottom). (**D**) Comparative functional analysis showing over-represented pathways involving proteins differentially expressed following platelet treatment with TRAP (left column), COLL (center column) or either compound (right column). (**E**) Scatter plots correlating changes in the expression of platelet proteins and the corresponding mRNAs in response to COLL (left, green) or TRAP (right, red). Dotted lines represent log2 fold-change (FC) cut-off.

To provide a comprehensive view of the platelet proteome, we performed high-resolution quantitative proteomic analysis on isolated and highly purified platelets from healthy volounteers (Fig. 1A and Supplementary Fig. 1A). In brief, a total of 15 platelet protein samples, pooled to obtain three biological replicates (five subjects for each pool/replicate), were subjected to MS analysis to perform accurate platelet proteome profiling. This allowed univocal identification and quantification of 5816 proteins (Supplementary Table 1A), providing the most comprehensive platelet proteome dataset obtained so far. *In silico* functional analysis of the proteome of resting platelets revealed, among the functions significantly represented, some related to mRNA translation and RNA processing, including splicing, such as mRNA processing and stabilization and processing of capped intron-containing pre-mRNA (Fig. 1B). Previous studies revealed the presence of functional spliceosome components within platelets^18^. Therefore, we next alligned the dataset obtained with the spliceosome network deposited in KEGG database finding, among the platelet proteins identified, 40 of the known components implicated in the major and/or minor splicing pathways^24^ according to Interactome database (Fig. 1C). Furthermore, splicing variant analysis was performed by using SpliceVista^25^ (Supplementary Table 2A,B), showing the presence of several protein isoforms. Proteome mapping was then carried out after *ex vivo* platelet activation with the known agonists Collagen (COLL) and Thrombin Receptor Activating Peptide (TRAP; Supplementary Fig. 1B) and differential analysis was performed to identify proteins specifically modulated upon activation. Confirming our previous results, obtained with a partial proteome view^13^, results showed several proteins differentially expressed upon activation (Supplementary Table 1B). In particular, considering |1.2| as fold-change cut-off, we found 259 modulated proteins by COLL (108 upregulated and 151 downregulated; Supplementary Table 1C) and 155 by TRAP (37 upregulated and 118 downregulated; Supplementary Table 1D). By merging the data, 172 and 78 proteins responded only to COLL (Fig. 2A) or to TRAP (Fig. 2B), while 88 showed a similar response to either stimulus (Fig. 2C). Interestingly, ca ∼20% consisted in each case of protein isoforms. Among the proteins commonly affected by either stimulus, 68 were downregulated and 17 upregulated, while only 3 were discordant. Moreover, comparative functional analysis (performed with Ingenuity Pathway Analysis tool (IPA), Qiagen) on the common, COLL-specific and TRAP-specific responsive protein sets revealed that these are specifically involved in signaling pathways highly related to platelet activity, e.g. acute phase response, atherosclerosis, coagulation and complement systems, although with significant differences among the three datasets (Fig. 2D). To investigate possible correlations between mRNA and protein responses to activating stimuli, we compared differentially expressed proteins with the corresponding transcripts identified by RNA-Seq. In this way, we found that changes in protein concentration following activation were often not supported by a comparable change of expression of the corresponding mRNAs, since none of these was statistically significant (FDR ≤ 0.05) and/or exceeded the fold-change cut-off (±1.5). This confirmed our previous observation^13^ that in activated platelets changes in protein expression often do not correlate with changes of the corresponding mRNAs (Fig. 2E).

### Detection of intron retention (IR) in resting platelets and induction of splicing by activating stimuli

**Figure 3:**
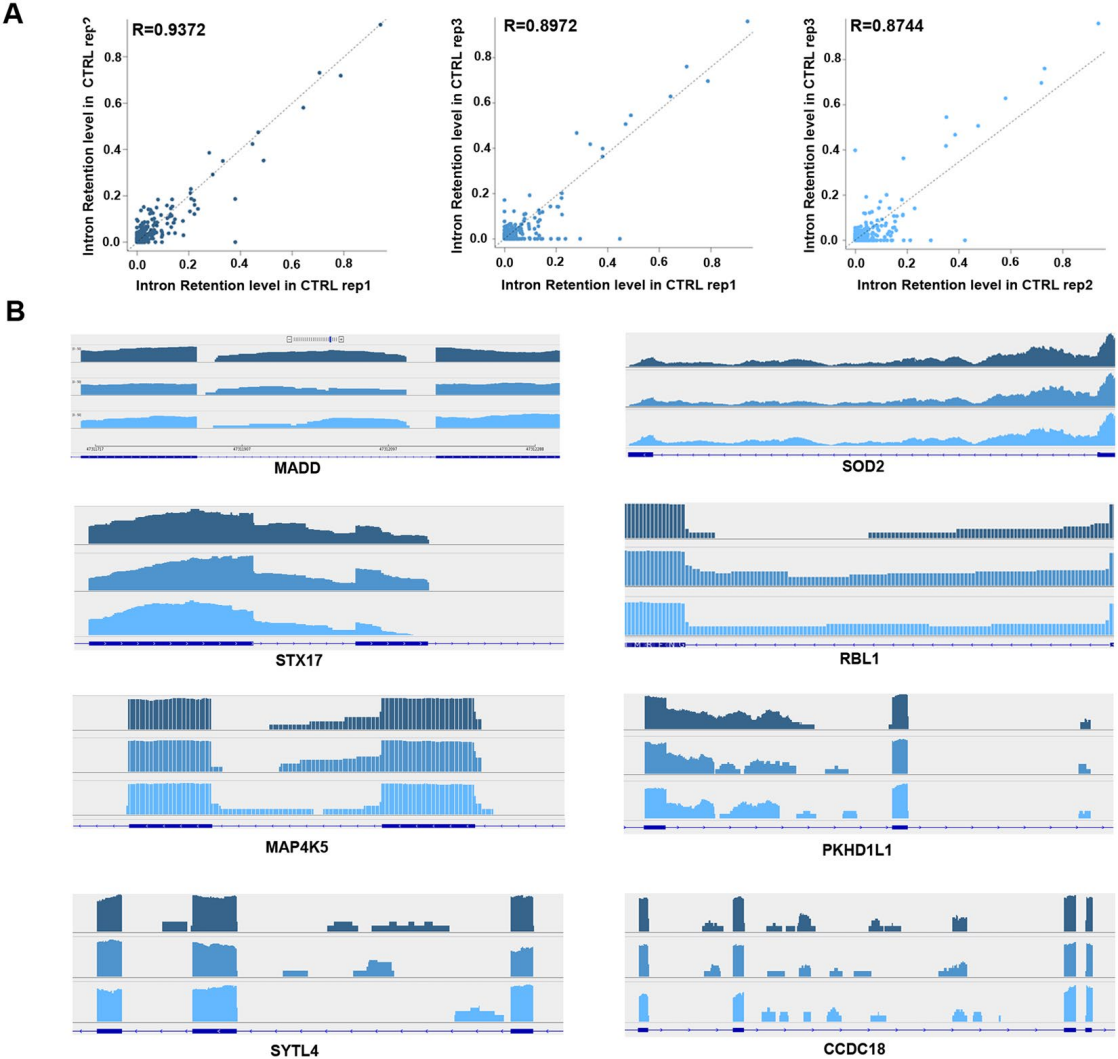
Intron retention analysis in the transcriptome of resting platelets. (**A**) Scatter plots showing pairwise correlation of IR events measured in biological triplicates. (**B**) IGV screen-shots displaying pre-mRNA with variable IR rates in resting platelets.

**Figure 4:**
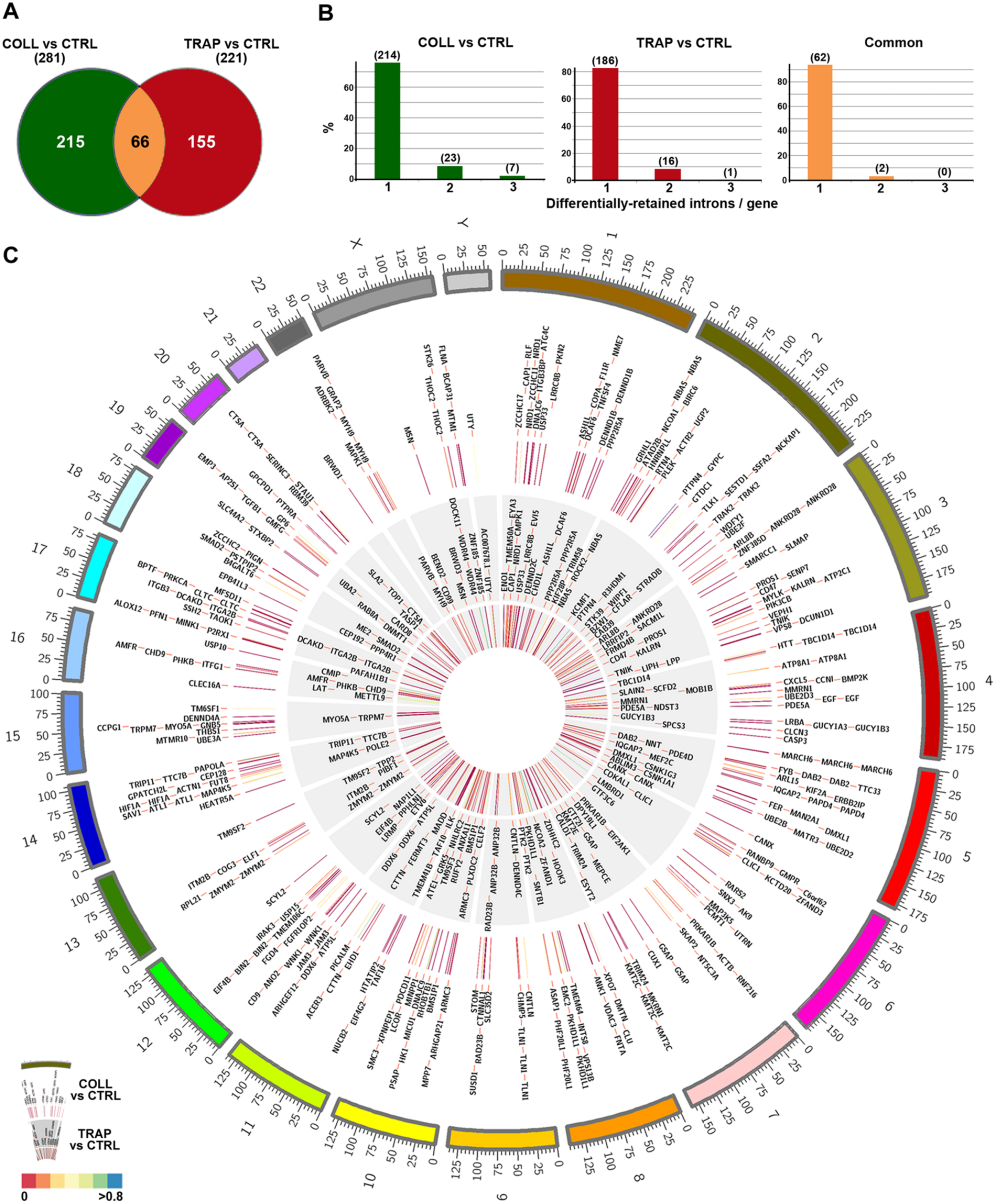
Differential intron retention analysis. (**A**) Venn reporting statistically significant downregulated (removed) introns following stimulation with COLL (green circle), TRAP (red circle) or either activator (orange). (**B**) Bar plots indicating the number of introns/gene showing significant IR ratio changes upon activation. Absolute frequencies are indicated above bars. (**C**) Circos plot showing statistically significant differentially retained introns after treatment with COLL (outer, white ring) or TRAP (inner, gray ring). Color dashes within circles represent the IR values for COLL (outer circle) and TRAP (inner circle), respectively. Most of the values fall near to 0 (orange-red), indicating intron reduction (loss) after platelet activation.

Given the evidence concerning the presence of components of the RNA splicing machineries in platelets^18^, we searched for partially spliced pre-mRNAs in resting platelets and assessed wether their maturation by intron removal occurs during platelet activation. To this aim, we analyzed RNAs isolated before and after platelet activation by strand-specific RNA-Seq, as described in the Methods section. This allowed the identification of a broad spectrum of platelet transcripts including, among others, mRNAs, lncRNAs and U snRNA components of the splicing machinery, confirming the possible presence of an active spliceosome in platelets (Table 1). Interesting, several pre-mRNAs were also found. Indeed, analyzing RNA-Seq data obtained under the same conditions with IRFinder^26^, we detected a total of 5628 statistically significant intron retention (IR) events, 3014 of which detected with high confidence in at least two of the three biological replicates investigated, deriving from 1426 genes (Supplementary Table 3A). In Supplementary Fig. 2 this result is summarized to show the trend of the 3014 introns in each replicate of resting platelets, represented in the circos plot as rings with different gray tones. The excellent correlation observed by pairwise comparisons of the three biological replicates (correlation factor close to 0.9 in each case) strongly supports the possibility that the IR transcripts identified did not result from random or casual events (Fig. 3A). We can exclude the possibility that the observed intron mapping reads derive from splicing lariats, since we searched within a list of circular RNAs containing introns in these samples and none of them overlapped the identified pre-mRNAs in resting platelets (data not shown). In Fig. 3B are reported some examples of pre-mRNAs with high to moderate IR rate, consistently identified in biological replicates of resting platelets, including STX17 (*Syntaxin 17*), SOD2 (*Superoxide Dismutase 2*) and SYTL4 (*Synaptotagmin Like 4*) pre-mRNAs, showing by RNA sequencing a highly, intermediate and poorly retained intron, respectively. By comparing these data with those relative to pre-mRNAs identified in agonist-treated platelets, 281 and 221 differentially retained introns were detected in RNAs from COLL- and TRAP-treated platelets, respectively (Supplementary Table 3B,C), among which 215 were removed to a significant extent after COLL and 155 after TRAP. By matching the two datasets, 66 introns, relative to 65 pre-mRNAs, were similarly affected by either treatment (Fig. 4A). Altogether, these data suggest that activation by different agonists induce intron removal from a sizeable number of immature platelet transcripts, concerning in most cases immature transcripts carrying in resting platelets only one intron, that was removed after activation (Fig. 4B), in line with the dynamic intron retention program described in the mammalian megakaryocyte lineage by Edwards *et al*.^27^. Graphic representation of introns differentially retained following COLL and TRAP treatment (circos plot in Fig. 4C) highlights how in most cases these undergo significant reduction after treatment, as shown by the color scale of the dashes, indicating IR ratios. Based on the function of the proteins encoded by these mRNAs, this phenomenon affects several of the pathways identified by comparative functional analysis of the differentially expressed protein sets, shown in Fig. 2D.

**Table 1:** snRNA components of pre-mRNA splicing machinery identified. List of U snRNAs expressed in CTRL, COLL and TRAP samples, detected by RNA-Seq. For each snRNA, average expression values of triplicate samples, indicated as FPKM (Fragments Per Kilobase Of Exon Per Million Fragments Mapped), are reported.

Ensembl_ID	Gene Name	CTRL (FPKM)	COLL (FPKM)	TRAP (FPKM)
ENSG00000201699	RNU1-59P	4.36	5.98	6.94
ENSG00000222985	RNU2-14P	2.51	3.27	0.00
ENSG00000222328	RNU2-2P	340.78	280.61	356.49
ENSG00000222414	RNU2-59P	2.48	3.41	7.26
ENSG00000223336	RNU2-6P	20.29	20.99	19.12
ENSG00000200795	RNU4-1	311.15	232.58	306.69
ENSG00000202538	RNU4-2	850.54	738.07	871.67
ENSG00000264229	RNU4ATAC	0.00	8.67	0.00
ENSG00000200156	RNU5B-1	36.93	0.00	0.00
ENSG00000238482	RNU6-1208P	0.00	0.00	74.14
ENSG00000221676	RNU6ATAC	320.49	140.90	154.62
ENSG00000270103	RNU11	16.15	61.66	41.98
ENSG00000270022	RNU12	287.64	281.31	322.20

In summary, these results demonstrate that platelet activation with two different physiological stimuli induces maturation of a significant number of immature transcripts present in resting platelets as a result of inefficient pre-RNA maturation in megakaryocytes.

### Validation of activation-induced splicing events by intersection of transcriptomics and proteomics data

**Figure 5:**
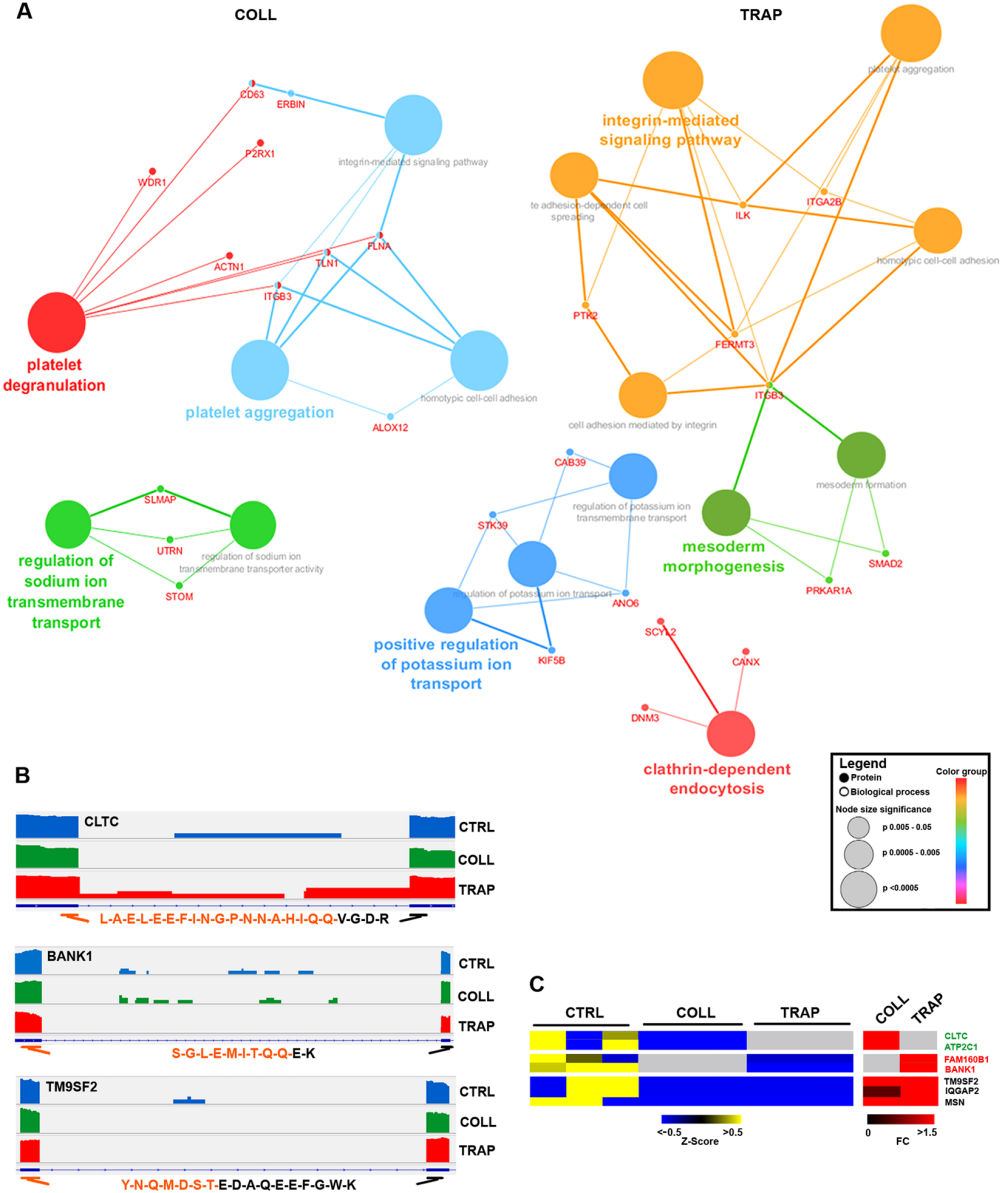
Correlations between intron removal and protein expression. (**A**) Functional networks showing pathways affected by upregulated proteins whose corresponding transcripts underwent intron removal upon platelet stimulation with COLL (left networks) or TRAP (right networks). (**B**) IGV screen-shots showing differentially retained introns in resting and activated platelets in transcript regions and the corresponding peptide sequence mapping on exon/exon junctions identified after activation. (**C**) Heatmap showing downregulated introns (yellow/blue heatmap) and the corresponding exon/exon junction peptide changes following activation (red heatmap) after activation with COLL and/or TRAP. Gray: not statististically significant.

To relate intron removal from pre-mRNAs to changes in platelet proteome composition during activation, we searched among the proteins found upregulated by either COLL or TRAP for those encoded by mRNAs found to maturate under the same conditions. Results revealed in several cases an inverse correlation between intron reduction and protein accumulation. Interestingly, while COLL induces maturation and translation of proteins involved in platelet degranulation and aggregation and in regulation of sodium ion transmembrane transport, TRAP mainly affects those involved in integrin-mediated signaling, clathrin-dependent endocytosis and positive regulation of potassium ion transport (Fig. 5A and Supplementary Tables 4 and 5). To further validate existing relationships between pre-mRNA maturation by intron removal and accumulation of mature proteins, we analyzed the MS data searching for peptide sequences mapping on exon/exon junctions (Supplementary Table 6A–C). We thereby found 208 peptides, 60% of which perfectly matched with RNA regions subject to intron removal. Searching the MS data for correlations between extent of intron removal and abundance of the resulting exon/exon junction peptide, we found that COLL activation specifically induced both events specifically on CLTC (*Clathrin Heavy Chain*) and ATP2C1 (*ATPase Secretory Pathway Ca2*+ *Transporting 1*), TRAP on FAM160B1 (*Family With Sequence Similarity 160 Member B1*) and BANK1 (*B-Cell Scaffold Protein With Ankyrin Repeats 1*) mRNAs and either stimulus on TM9SF2 (*Transmembrane 9 Superfamily Member 2*), IQGAP2 (*IQ Motif Containing GTPase Activating Protein 2*) and MSN (*Moesin*) gene products (Fig. 5B,C). As an example, in Fig. 5B are reported Intergrative Genomics Viewer (IGV) screen-shots visualizations of CLTC, BANK1 and TM9SF2 mRNAs, showing the extent of intron removal following COLL, TRAP or either stimulus, respectively, and the petides that mapped in the corresponding exon/exon junctions shown by MS to accumulate in each case (Fig. 5C). When considering the technical limitation of the MS technology, in particular its relatively low sensitivity for detection of such peptides, these results provide a confirmatory evidence that pre-mRNA maturation promoted in platelets by activating stimuli results in accumulation of a specific set of proteins encoded by these RNAs.

## Discussion

Platelets play a central role in the hemostatic process and, although being small cytoplasmic fragments, they are metabolically active as they contain several functionally active organelles and are equipped with an elaborate intracellular machinery. Indeed, platelets are able to syntethize a limited amount of proteins starting from resident mRNAs. Several studies have shown that monitoring protein profiles can be useful for evaluation of drug responses^28–30^ and for this reason platelet proteome characterization under resting and activated state can provide useful information. Indeed, understanding proteome changes following platelet activation can help find new ways to diagnose, monitor and treat diseases caused by platelet dysfunctions. It is now clear that platelet proteome can be reorganized through post-transcriptional/translational processes and, among these, cytoplasmic splicing has been proposed as a regulatory mechanism active during platelet activation^18,31^. Recent evidences indicate that IR-induced mRNA modulation is fairly common and represents a biologically relevant process, active during hemopoietic lineage maturation^27^. Although IR is typically associated with mRNA degradation, it can also influence protein production. In fact, it has been demonstrated that transcripts encoding for tissue factor and IL-1β that carry retained introns are present in megakaryocytes and are carried over into anucleate platelets, where introns are spliced out upon platelet activation^18,19^. Starting from this evidence, we combined proteome and whole transcriptome profiling data generated before and after platelet activation, by applying a proteogenomics approach to search for evidences of cytoplasmic splicing events occurring in activated paltelets and influencing composition of the proteome. To this aim, we first applied HiRIEF coupled to LC-MS to characterize the platelet proteome in depth before and after activation by two physiological stimuli. This allowed us to widen the number of platelet proteins identified so far and led to the identification of several proteins whose level is modulated by the activation, including specific isoforms. Recently, a platelet proteome analysis performed by Lee *et al*. before and after antiplatelet administration^32^ led to the identification of ~5400 proteins, a comparison of this dataset with the one reported here showed about 62% overlap, indicating that a combination of the two datasets now covers the entire platelet proteome, as predicted from transcriptome analysis that detected ~8000 protein-coding RNAs (Cimmino *et al*.^13^ and present study). On the other hand, IR analysis revealed a very significant number of immature RNAs, many of them being matured during the activation process. We were able to provide a proof of concept that in several cases this may lead to protein accumulation, as we found inverse correlation between IR and protein, or even exon/exon junction peptide, concentration. In particular, we demonstrated intron removal coupled to exon/exon peptide accumulation for CLTC and ATP2C1 in response to COLL, FAM160B1 and BANK1 in response to TRAP and TM9SF2, IQGAP2 and MSN in response to either activator (Fig. 5C). Interestingly, these proteins are involved in key processes responsible for the shape changes occurring during platelet activation, such as cytoskeleton remodeling and calcium mobilization. Indeed, the involvement of clathrin in vesicle trafficking and cell migration it is well known^33^ and the COLL-modulated clathrin heavy chain (CLTC) has been shown to be directly involved in the control of hemostasis and platelet responses^34^. Similarly, IQ Motif Containing GTPase Activating Protein 2 (IQGAP2) regulates cell motility and morphology by interacting with cytoskeleton components and it has been demonstrated to be an effector of thrombin-induced platelet cytoskeleton remodeling^35^. On the other hand, Moesin (MSN) has been described to induce platelet shape change *via* Rho signaling^36^ and to be a binding partner of the adhesion molecule PECAM-1 (*Platelet And Endothelial Cell Adhesion Molecule 1*) in activated but not in resting platelets^37^, while ATPase Secretory Pathway Ca2+ Transporting 1 (ATP2C1), B-cell scaffold protein with ankyrin repeats 1 (BANK1) and transmembrane 9 superfamily member 2 (TM9SF2) are involved in ion transport and calcium mobilization^38–40^, a crucial event determining granule secretion during platelet activation^40,41^. Overall, these results suggest a biological significance endowed in induction of resident pre-mRNA maturation during platelet activation, promoting selective changes of the platelet proteome by neo-synthesis of proteins involved in platelet shape changes, and possibly other key processes, during thrombosis.

In conclusion, together with other several processes, such as miRNAs expression modulation^13^, extensive maturation of resident pre-mRNAs occurs in platelets in response to activating stimuli, representing a mechanism for post-transcriptional control of proteome composition in these anucleated cell fragments. In the present work we investigated this hypothesis after excluding other possible events mimicing pre-mRNA variations. Indeed, RNA-Seq reads mapping on retained introns were not associated to either nc- or anti-sense RNAs, since the vast majority of them was assigned to protein coding transcripts and, more important, the use of a strand-specific protocol for library preparation prevented ambiguities due to the presence of anti-sense RNAs. In addition, finding among the identified RNAs also U snRNA components of the splicing machinery reinforced the evidence that this was active. Quantitation of these events could provide a useful parameter to evaluate the extent of platelet activation under different physio-pathological conditions, while a better understanding of the mechanism(s) controlling this process might led to new therapeutic approaches to prevent, or control, platelet activation in specific pathological conditions, similar to what was recently proposed for glioblastoma and possibly other cancers^21,22^. In this respect, some of the signal transduction pathways involved in platelet response to activating stimuli^42^ overlap with those known to control splicing and to be amenable to pharmacological modulation^43^.

## Methods

### Platelet purification and activation

Fifteen healthy volunteers (all males with no cardiovascular risk factors), who refrained from taking any medication, were enrolled. A written informed consent was obtained from each subject. The protocol was approved by the local ethical committee of the University of Campania “Luigi Vanvitelli” and all experiments were performed in accordance with relevant guidelines and regulations. Fifty ml of blood from each donor were collected into 0.105 M sodium citrate tubes (1:10 V/V) and processed within 15 min from sampling. For platelet isolation, blood samples were centrifuged at 160 × g for 15 min at room temperature. The platelet-rich plasma obtained was then filtered using a specific leukocyte removal system (Pure Cell PALL, Pall Italia), as described earlier^13^. Leukocyte contamination was assessed by flow cytometry and rtPCR analysis of leukocyte (CD-45) and platelet (CD-41) specific antigen mRNA, with GAPDH RNA used as control, as described in Supplementary Information (Methods section). Purified platelets from each donor were split in three aliquots: a first aliquot was immediately processed and used as control (baseline: T0), two aliquots were treated with Collagen (COLL, 60 µg/mL) or Thrombin Receptor Activating Peptide (TRAP 25 µM) under continuous stirring for 120 minutes at 37 °C before processing. Platelet aggregation was verified in all samples before and after stimulation by the formation of macroaggregates and confirmed by light transmission aggregometry as described in Supplementary Information (Methods section). After two hours of stimulation, platelets were centrifuged at 2,000 × g at 4 °C, washed three times with PBS and then stored at -80 °C until further processing.

### RNA and protein extraction

Purified platelets from each of the 15 donors were subjected to total RNA and protein extraction using the mirVana PARIS kit (Thermofisher scientific) according to the manufacturer’s instructions. 600 µl of ice-cold Disruption Buffer from the kit were added to each platelet aliquot (the 15 donors) and 3 pools of 5 samples/each were made. One third of each pool was withdrawn for protein extraction, carried out as described^13^, and the remaining was processed for RNA purification as described earlier^13^. RNA concentration in each sample was assayed with a ND-2000c spectrophotometer (Thermo Scientific) and its quality and integrity assessed with the Agilent 2100 Bioanalyzer with Agilent RNA 6000 pico kit (Agilent Technologies). Protein concentration was determined using Bradford Protein Assay and EZQ Protein Quantitation Kit. Three independent biological replicates for each treatment and for the CTRL were used for all subsequent analyses.

### Mass Spectrometry and data analysis

#### Sample preparation

Protein sample preparation for MS analyses was performed as previously described^23^. In brief, samples were cleaned, resuspended and sonicated in a buffer containing 4% SDS, 25 mM HEPES pH 7.6 and 1 mM DTT. Total protein amount was estimated (Bio-Rad DC). For filter aided sample preparation, 150 µg of protein sample was mixed with 1 mM DTT, 8 M urea, 25 mM HEPES pH 7.6 in a centrifugation filtering unit with a 10 kDa cut-off (Nanosep® Centrifugal Devices with Omega™ Membrane, 10 k). The samples were then centrifuged for 15 min, 14.000 g, followed by another addition of the 8 M urea buffer and centrifugation. Proteins were alkylated by 25 mM IAA, in 8 M urea, 25 mM HEPES pH 7.6 for 10 min, centrifuged, followed by two more additions and centrifugations with 8 M urea, 25 mM HEPES pH 7.6. Protein samples were digested on the filter using trypsin (sequencing grade modified, Promega, enzyme:protein ratio 1:50), in a buffer containing 0.25 M urea, 50 mM HEPES overnight at 37 °C. After digestion, the filter units were centrifuged for 15 min, 14.000 g, followed by another centrifugation with MilliQ water. Peptides were collected and the peptide concentration determined. Before labelling, the pH of the samples was adjusted using TEAB pH 8.5 (30 mM, final concentration). The resulting peptide mixtures were labelled with TMT 10 plex (Thermo Scientific) according to manufacturer’s instructions and then pooled. Labelling efficiency was determined, before pooling, by LC-MS/MS by running short (15 min) gradients of all samples after labelling with an Agilent HPLC coupled to a Velos, run with CID and HC, data were then searched in a simple workflow (SEQUEST, no Percolator) with TMT-labelling set as dynamic, to verify that labelling efficacy was >99% at the PSM level. Sample clean-up was performed by solid phase extraction (SPE strata-X-C, Phenomenex) and purified samples were dried in a SpeedVac.

#### High Resolution IsoElectric Focusing (HiRIEF)

After clean-up the sample pools were subjected to peptide IEF-IPG (isoelectric focusing by immobilized pH gradient) in pI range 3–10. Dried peptide samples (300 µg) were dissolved in 160 µL rehydration solution containing 8 M urea, and allowed to adsorb to the gel bridge by swelling overnight. The 24 cm linear gradient IPG strips (GE Healthcare) were incubated overnight in 8 M rehydration solution containing 1% IPG pharmalyte pH 3–10 (GE Healthcare). Samples were applied to the IPG strips by the gel bridge (pH 3.7) at the cathode end and run as described^44,45^. After focusing, the peptides were passively eluted into 72 contiguous fractions with MilliQ water using an in-house constructed IPG extractor robotics (GE Healthcare Bio- Sciences AB, prototype instrument) into a 96-well plate (V-bottom, Corning product #3894), which was then dried in a SpeedVac. The resulting fractions were freeze dried and kept at −20 °C.

#### LC-ESI-MS/MS

Online LC-MS was performed using a hybrid Q-Exactive mass spectrometer (Thermo Scientific), connected to a UPLC 3000 systen (Dionex). For each LC-MS/MS run, the auto sampler dispensed 8 μl of solvent A to the well in the 96 V plate, mixed for 10 min, and proceeded to inject 3 μl. FTMS master scans with 70,000 resolution (and mass range 300–1700 m/z) were followed by data-dependent MS/MS (17,500 resolution) on the top 5 ions using higher energy collision dissociation (HCD) at 30% normalized collision energy. Precursors were isolated with a 2 m/z window. Automatic gain control (AGC) targets were 1e6 for MS1 and 1e5 for MS2. Maximum injection times were 100 ms for MS1 and 500 ms for MS2. The entire duty cycle lasted ~2.5 s. Dynamic exclusion was used with 60 s duration. Precursors with unassigned charge state or charge state 1 were excluded. An underfill ratio of 1% was used.

#### Peptide and protein identification and quantification

The MS raw files were searched using SEQUEST and the output was further refined by Percolator on the software platform Proteome Discoverer 1.4 (Thermo) against the human Uniprot database (90440 entries, including 23204 isoforms) and filtered to a 1% FDR cut off. A precursor ion mass tolerance of 10 ppm, and product ion mass tolerances of 0.02 Da for HCD-FTMS was used. The algorithm considered tryptic peptides with maximum 1 missed cleavage; carbamidomethylation (C), TMT 10-plex (K, N-term) as fixed modifications; oxidation (M) as variable modification. Quantification of reporter ions was done by Proteome Discoverer on HCD-FTMS tandem mass spectra using an integration window tolerance of 20 ppm. Only unique peptides in the data set were used for quantification.

#### Data analysis

To identify statistically modulated proteins a two-samples t-test was applied using on Log2 transformed median expression value of the three biological replicates of COLL and TRAP treated *vs* CTRL samples with a p-value computed on permutation (10000 permutations) and an alpha 0.01^46^. A further Bonferroni’s correction was then applied. Protein identification and quantification data are reported in the Supplementary Table 1A–D. Splicing variant analysis was performed by using SpliceVista^25^ and results are reported in Supplementary Table 2A,B. Finally, for exon-exon junction peptide identification a proteogenomics approach was carried out by searching peptide spectra in a customized database including *in silico* translated peptide sequenced spanning splice junctions from retained introns detected by total RNA-seq data analyses in CTRL samples. To generate the customized database, the nucleotide sequences were extracted ±75 bp from genomic coordinates of junction sites and were translated in three reading frames to amino acid sequences. Results obtained are reported in Supplementary Table 6A–C.

### RNA sequencing and data analysis

#### RNA-Seq

Indexed libraries were prepared using 250 ng of total RNA as starting material, with TruSeq Stranded Total RNA Sample Prep Kit (Illumina Inc.). Libraries were sequenced (paired-end, 2 × 100 cycles) at a concentration of 8 pM/lane on HiSeq2500 platform (Illumina Inc.) and analyzed as previously described^13^.

#### Data analysis

The raw sequence files generated (.fastq files) underwent quality control analysis using FASTQC (http://www.bioinformatics.babraham.ac.uk/projects/fastqc/) and the quality checked reads were then aligned to the human genome (assembly hg38) using STAR version 2.5.0a^47^. Differentially expressed transcripts (Fold-change ≥ |1.5|, FDR ≤ 0.05) were identified as described^48^.

Intron retention level (IR ratio) was computed for each sample using IRFinder tool^26^ with standard parameters. IRFinder excludes features that overlap with introns such as microRNA or snoRNAs as these may confound the accurate measurement of true intron levels.

IR ratio is calculated as follows:

$$\frac{intronic\,abundance}{(intronic\,abundance+exon\,splice\,abundance)}$$

For each sample, IR ratio greater than first quantile of its distribution has been considered for further analysis. In addition, only introns showing IR ratio in at least two out of three biological replicates were considered for further analysis.

Statistically significance of differentially retained introns among COLL and TRAP *vs* CTRL samples were assessed appling two-sample t-test with 10000 permutations and appling a FDR cut-off of 0.01.

Functional analysis was performed using ClueGO^49^. Circos plot were generated using Clico^50^.

### Statistical analyses

Proteomics experiments were performed on three biological replicates. The MS raw files were searched using the software platform Proteome Discoverer 1.4 (Thermo) against the human Uniprot database and filtered to a 1% FDR cut off. To identify statistically modulated proteins a two-samples t-test was applied using Log2 transformed median expression value of the three biological replicates of COLL and TRAP treated vs CTRL samples with a p-value computed on permutation (10.000 permutations) and an alpha cutoff of 0.01. A further Bonferroni’s correction was then applied. Proteins were considered differentially expressed if they showed a fold-change cutoff |1.2|. RNA-Seq analyses, were performed using three biological replicates. RNAs were considered to be expressed when they were detected with an expression value ≥10 reads. Differentially expressed RNAs were computed using DESeq2^51^, considering a fold-change cut-off |1.5| and a FDR ≤ 0.05. For intron retention analysis, an intron was considered retained with an expression value above the first quantile of the expression distribution level for the considered dataset/replicate and if it was retained in at least two/three replicates. To determine differentially retained introns a two-samples t-test was applied. p-value was computed on permutation (10.000 permutations) with an alpha cutoff of 0.01. A further Bonferroni’s correction was then applied. For Gene ontology analyses, only functional cathegories with a FDR ≤ 0.05 were considered. For IPA analyses, only expressed genes (detected by R NA-Seq) within platelets were used as background.

### Accession codes

Raw MS data have been deposited to the ProteomeXchange Consortium via the PRIDE^52^ partner repository, with dataset identifier PXD006559. Raw RNA-Seq data are available in EBI ArrayExpress with Accession Number E-MTAB-6148.

## Electronic supplementary material

Supplementary Information

Supplementary Table 1

Supplementary Table 2

Supplementary Table 3

Supplementary Table 4

Supplementary Table 5

Supplementary Table S6
