# Supplementary material for: Splicing of platelet resident pre-mRNAs upon activation by physiological stimuli results in functionally relevant proteome modifications
Source: Sci Rep. 2018 Jan 11;8:498. doi: 10.1038/s41598-017-18985-5 (PMC5765118; doi:10.1038/s41598-017-18985-5)
Supplement: Supplementary file 1 — Supplementary Information [file 41598_2017_18985_MOESM1_ESM.pdf]

Splicing of platelet resident pre-mRNAs upon activation by physiological stimuli results in functionally relevant proteome modifications

Giovanni Nassa[1§], Giorgio Giurato[1,2§], Giovanni Cimmino[3], Francesca Rizzo[1], Maria Ravo[1,2], Annamaria Salvati[1], Tuula A. Nyman[4], Yafeng Zhu[5], Mattias Vesterlund[5], Janne Lehtiö[5], Paolo Golino[3], Alessandro Weisz[1*] and Roberta Tarallo[1*]

[1]Laboratory of Molecular Medicine and Genomics, Department of Medicine, Surgery and Dentistry "Scuola Medica Salernitana", University of Salerno, Baronissi (SA), Italy

[2]Genomix4Life srl, Department of Medicine, Surgery and Dentistry "Scuola Medica Salernitana", University of Salerno, Baronissi (SA), Italy

[3]Department of Cardio-Thoracic and Respiratory Sciences, Section of Cardiology, University of Campania "Luigi Vanvitelli", Naples, Italy

[4]Department of Immunology, Institute of Clinical Medicine, University of Oslo and Rikshospitalet Oslo, Oslo, Norway

[5]Science for Life Laboratory, Department of Oncology-Pathology, Karolinska Institutet, Stockholm, Sweden

[§] These authors contributed equally to this work

[*] Corresponding authors

MATERIAL AND METHODS

SUPPLEMENTARY FIGURES 1-2

FIGURE LEGENDS

REFERENCES

METHODS

Evaluation of platelet purity

Platelet purity was assessed by flow cytometry and amplification of CD41 and CD45 by real time-PCR using the following primers: CD41: Fw GAA TCG CGA TGT TGG TGA GC and Rev GGC TGG AAA GGA GTT CCC TC; CD45: Fw GTG AGA GTG GAC GAT AAA GGG A; Rev TTC TGG TGT CTG CCT GCT TC.

Evaluation of Platelet aggregation

The level of purified platelet aggregation was measured with light transmission aggregometry (LTA) in PRP, following activation by TRAP (25µM) and COLL (60µg/mL), at 37°C following standard procedure[S1].

Supplementary Figure 1

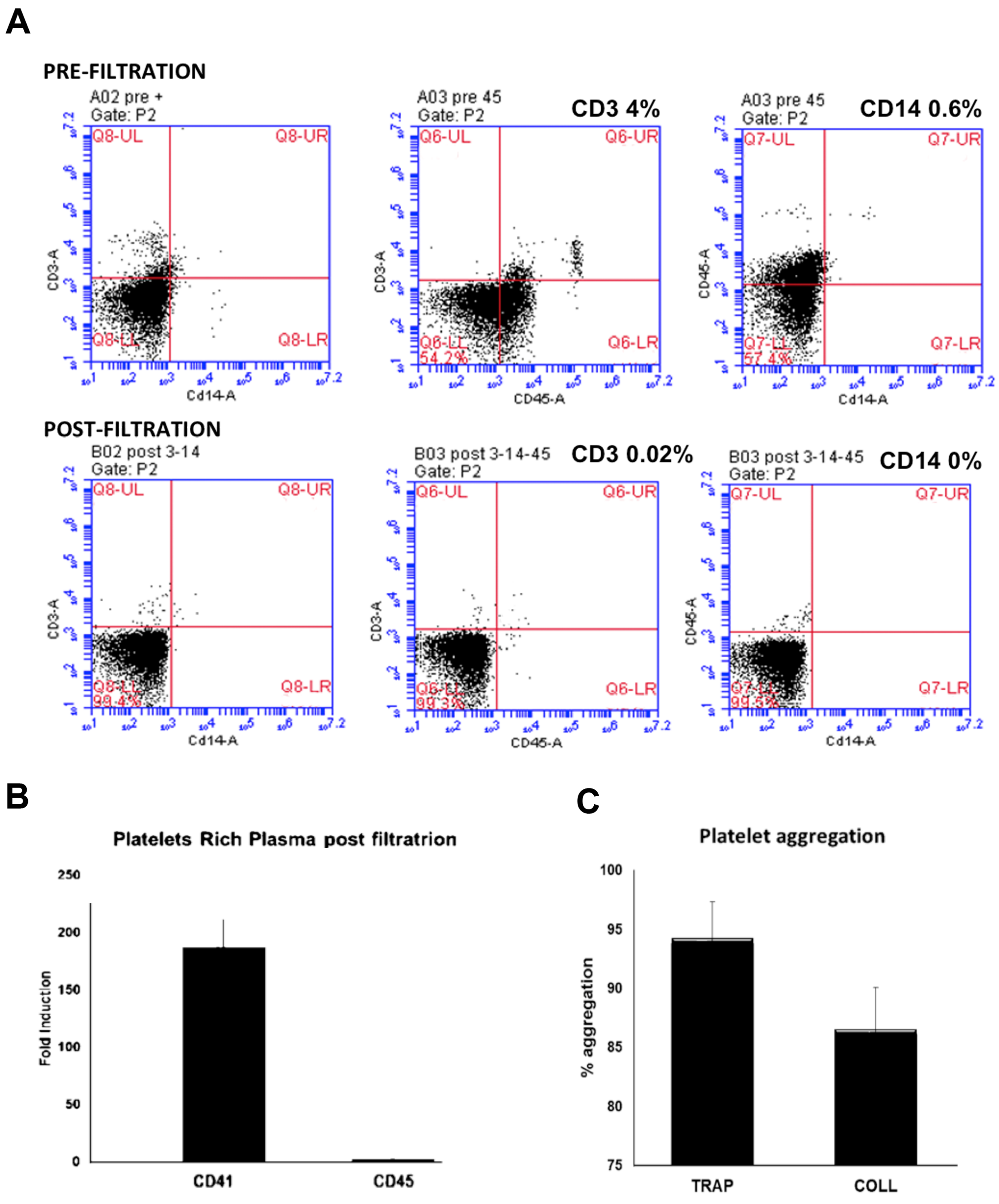

Supplementary Figure 2

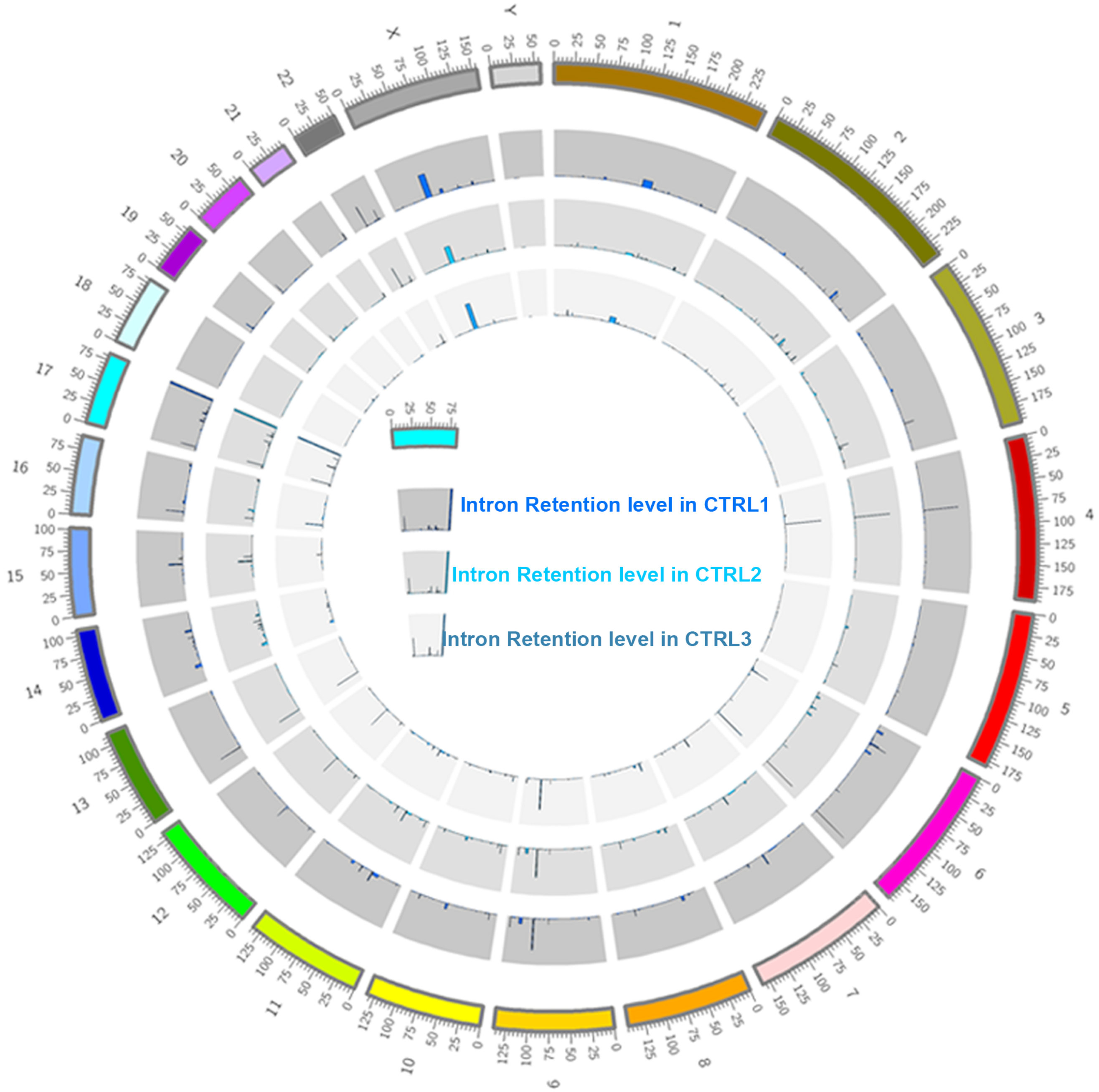

FIGURE LEGENDS

Supplementary Figure 1. Assessmnet of pletelet preparations purity and *ex vivo* aggregation following COLL or TRAP activation. (A) Platelet purification and flow cytometry analysis. Purity of the platelet preparation was controlled by flow cytometry. Leukocyte contamination (CD3 and CD 14) was assayed before and after purification. Filtration reduced the amount of CD3 and CD14 positive cells from 4% and 0.6% to 0.02% and 0% respectively. **(B) RT-qPCR analysis of leucocyte-specific CD45 and platelet-specific CD41 mRNA level.** Platelet purity was checked after total RNA extraction, cDNA systhesis and qPCR. Data shown indicate the relative expression of leukocyte-specific CD45 mRNA and platelet-specific CD41 mRNA level with respect to GAPDH used as control. **(C) Determination of platelets aggregation.** Activating stimuli induced rapid platelet aggregation, measured with light transmission aggregometry (LTA): TRAP 94.2±3.11%, COLL 86.5±3.53%.

Supplementary Figure 2. Intron Retention (IR) analysis. Circos plot showing retained intron levels, measured with IRFinder, among the expressed transcripts identified by RNA-Seq in resting platelets. Triplicate samples are represented as rings with different grey tones. The more high are the bars, the more retained are the corresponding introns.

REFERENCES

S1. Hayward CP, Moffat KA, Pai M, Liu Y, Seecharan J, McKay H, et al. An evaluation of methods for determining reference intervals for light transmission platelet aggregation tests on samples with normal or reduced platelet counts. Thrombosis and haemostasis. 2008;100(1):134-45.
